# Using genotyping to delineate tuberculosis transmission in long-term care facilities: single facility 4-year experience

**DOI:** 10.1186/s12879-017-2526-2

**Published:** 2017-06-13

**Authors:** Wen-Cheng Chao, Pei-Chun Chuang, Don-Han Wu, Chieh-Liang Wu, Po-Yu Liu, Chi-Chang Shieh, Ruwen Jou

**Affiliations:** 1 Department of Medical Research, Taichung Veterans General Hospital, Taichung, Taiwan; 2 Institute of Clinical Medicine, National Cheng Kung University Medical College, Tainan, Taiwan; 3 Reference Laboratory of Mycobacteriology, Tuberculosis Research Center, Centers for Disease Control, No.6, Linsen S. Rd., Jhongjheng District, Taipei City, 10050 Taiwan; 4 Department of Internal Medicine, Taichung Veterans General Hospital Chiayi branch, Chiayi, Taiwan; 5 Department of Internal Medicine, Taichung Veterans General Hospital, Taichung, Taiwan; 6 Center for Quality Management, Taichung Veterans General Hospital, Taichung, Taiwan; 7 Institute of Microbiology and Immunology, National Yang-Ming University, Taipei, Taiwan

**Keywords:** Tuberculosis, Genotyping, Transmission, Long-term care facility, Outbreak

## Abstract

**Background:**

Residents in long-term care facilities (LTCFs) are vulnerable to tuberculosis (TB) transmission; however, to delineate possible routes of TB transmission in LTCFs is difficult. This study aimed to address the use of regular genotyping surveillance to delineate TB transmission in LTCFs.

**Methods:**

All of *Mycobacterium tuberculosis* isolates in the reported 620-bed LTCF between July 2011 and August 2015 were genotyped, and we retrospectively compared epidemiological data and genotyping results.

**Results:**

A total of 42 subjects were diagnosed with culture-positive pulmonary TB infection during the 4-year period. Their median age was 76.5 years, and 64.3% (27/42) of them were male. Genotyping identified 5 clustered TB infections involving 76.2% (32/42) of all TB subjects. In a multivariate logistic regression model adjusted for age, sex, chronic obstructive pulmonary disease, and body mass index, subjects with clustered TB infection were less likely to be Activities of Daily Living (ADL)-dependence (adjOR 0.073, 95% CI 0.007–0.758) when compared with subjects having individual TB infections. Prolonged surveillance is essential given that the median interval to diagnose secondary subjects was 673 days. Finally, only 63.0% (17/27) of the 27 secondary TB subjects in this study had contact history with index subject in the same ward.

**Conclusions:**

In conclusion, possible routes of TB transmission in a complex TB outbreak at LTCFs might be delineated by routine genotyping surveillance and regular health check-up.

**Electronic supplementary material:**

The online version of this article (doi:10.1186/s12879-017-2526-2) contains supplementary material, which is available to authorized users.

## Background

Long-term care facilities (LTCFs), providing medical care for the elderly or disabled individuals resulting from chronic diseases including mental illness, are vulnerable to tuberculosis (TB) transmission because of the enclosed environment in LTCFs [1, 2]. TB in the elderly is now a worldwide public health concern with 52.7% (25,199/47821) of newly diagnosed TB patients between 2010 and 2014 in Taiwan were those aged more than 65 years [3–5]. However, to delineate the possible route of transmission of TB among residents in LTCFs based on the epidemiological investigation is somehow difficult because of the potential false-positive epidemiological correlation among residents with a high incidence of TB disease [2, 6]. Genotyping surveillance is increasingly used to be a pivotal tool in the investigation of TB outbreak not only to confirm TB transmission among those with suspected epidemiological relationships [7] but also to identify previously unrecognized TB transmissions [8–11]. The goal of this study was to delineate possible routes of TB transmission in a 620-bed LTCF and to investigate factors associated with clustered TB infection using the 4-year clinical follow-up and genotyping surveillance data.

## Methods

### Setting

The reported LTCF is located in southern Taiwan and consists of a 300-bed general healthcare section (A) majorly caring the elderly and a 320-bed healthcare section (B) caring individuals with mental illness. There were 5 wards in section A and 8 wards in section B, while no contact activities exist between residents living in different sections. However, 2 of the 8 wards in section B shared the same ventilation system with wards in section A. The daily activity varied in these 2 sections because of the different characteristic of residents. The daily activity of those with mental illness living in section B was mostly restricted in their wards, but supervised group activities including group psychotherapy and vocational rehabilitation were allowed. The activity of those who lived in section A was unrestricted; however, most of the residents were the elderly in Activities of Daily Living (ADL) dependent status. Barthel Index is officially used to assess the dependence of ADL in Taiwan, while Barthel Index score equal or lower than 20 is defined as total dependence [12]. In this study, ADL-dependence was defined as subjects whose Barthel Index scores was equal or lower than 20.

### Study population

In this retrospective study, active case finding was suggested and supervised by Taiwan Centers for Disease Control (Taiwan CDC) after the first TB clustered infection identified in 2011. Therefore, all residents living in the reported facility had received regular chest X-ray (CXR) follow-up and sputum mycobacteriology check-up every six months [13]. Both liquid media system (Becton and Dickinson, USA) and solid (Middlebrook 7H11) media were used for sputum cultures. Additionally, all newly diagnosed TB subjects from July 2011 to August 2015 were assessed, and their medical records were reviewed to obtain demographic and clinical information regarding TB for each patient. In the reported facility, the test for human immunodeficiency virus (HIV) is one of routine admission examinations for all residents, and all of the subjects in this study are negative for HIV. This study was approved by the Institutional Review Board of Taichung Veteran General Hospital (CG16079B). Written informed consent for participants’ clinical records to be used in this study was waived, and patient information was anonymized and de-identified before analysis.

### Genotyping

All culture-positive *Mycobacterium tuberculosis (M. tb)* isolates in the reported facility after 2011 were sent to Taiwan CDC for genotyping. Combined restriction fragment length polymorphism (RFLP) of the insertion sequence IS6110 and spacer oligonucleotide typing (spoligotyping) analysis was used for genotyping interpretation between 2011 to 2014-Nov. Spoligotypes were compared with the SITVIT global database [14]. Given that mycobacterial interspersed repetitive units-variable number tandem repeats (MIRU-VNTR) is more convenient for comparison with databases at different laboratories [15], the 10-locus MIRU-VNTRwas added on the RFLP-spoligotyping for genotyping in 2014-Nov. Briefly, the similarity of patterns was calculated by the unweighted pair group method with arithmetic averages (UPGMA) and Dice similarity coefficient [16]. Of the 37 isolates analyzed by RFLP and spoligotyping before 2014-Nov., 22 representative isolates were further analyzed by 10-locus MIRU-VNTR for comparison. Additionally, the clustered isolate was defined as two or more than two subjects with *M. tuberculosis* isolates having identical genotypes, and each cluster was assigned a unique cluster number for these matched isolates, whereas isolates without matched genotyping pattern were defined as individual isolates.

### Investigation of epidemiologic links

Patients whose isolates had matched patterns of genotyping were considered to be subjects of the clustered TB infection. Index subject of each cluster was defined as the first patient who was diagnosed with TB disease, while other patients in the same cluster were considered to be secondary subjects. Positive epidemiologic links were defined as patients in a cluster having contacts in the same ward for cumulatively more than 40 h [17]. If the investigation did not yield any of the above links, patients were classified as not having an epidemiologic link.

### Statistics

Data were presented as frequencies (n) or percentages (%) for categorical variables and as median (interquartile range) for continuous variables. Differences between clustered TB subjects and non-clustered TB subjects were evaluated by Mann-Whitney test for continuous variables and Fisher exact test for categorical variables. A multivariate logistical regression model was used to identify variables that correlated with TB cluster-infection after controlling age, sex, and other significant variables (*P* < 0.20) in univariate analysis. Statistical significance was set at *P* < 0.05, two-sided. All data were analyzed using SPSS version 23.0 (SPSS Inc., Chicago, IL, USA).

## Results

### Characteristics of enrolled subjects

**Fig. 1:**
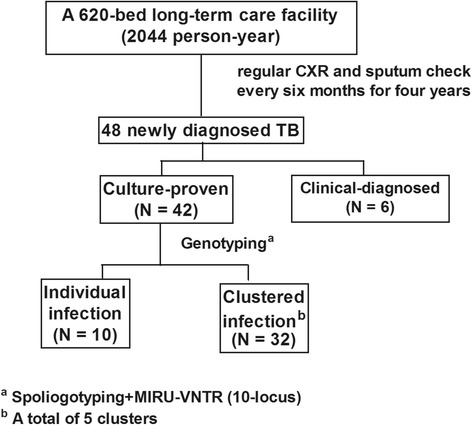
Participant enrollment flowchart

**Fig. 2:**
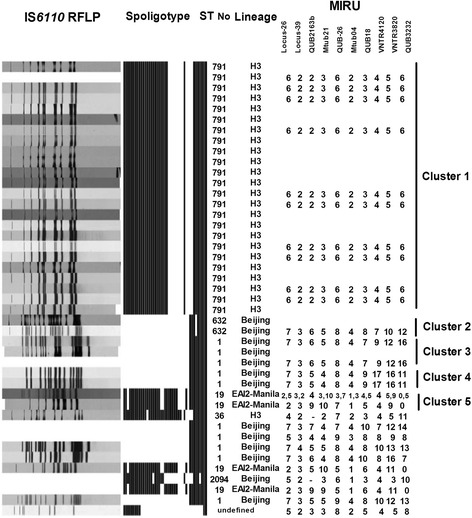
Genotyping analyses of all culture-proven participants with pulmonary TB infection

A total of 2044 person-year was surveyed, and 48 subjects were diagnosed with TB disease during the 4-year period. Of the 48 subjects, 87.5% (42/48) had a positive sputum mycobacterial culture (Fig. 1). Of the 6 clinically diagnosed TB subjects, 1 (16.7%) was diagnosed by caseous granuloma in lung biopsy, 1 (16.7%) by high pleural adenosine aminohydrolase (ADA) (106 IU/mL) level, and 4 (66.7%) by serial image findings. All of *M. tb* isolates from the 42 culture-proven TB subjects were sent to the central laboratory of Taiwan CDC for genotyping, and the clinical characteristics of the 42 TB subjects are summarized in Table 1. Their median age was 76.5 years, and 64.3% (27/42) of them were male. Thirty-two subjects (76.2%) were categorized as clustered TB infection according to results of genotyping, whereas 10 (23.8%) subjects were considered to be individual TB infections. Of the 42 culture-positive isolates genotyped, 15 *M. tb* strains were identified in this study. Five strains were attributed to clustered TB infection, while the other 10 strains were individual TB infection. There were 23 patients in cluster-1, and the number of patients in the cluster-2, −3, −4 and −5 were 2, 3, 2, and 2, respectively. One patient in cluster-5 was mixed infection which can be proved by the consistent results of RFLP and MIRU-VNTR. The major pattern of RFLP and all MIRU loci besides QUB2163b was the same between the two strains in cluster-5. Therefore, strains having one-locus difference and epidemiological links in this study were considered as one cluster. In line with other reporters investigating *M. tb* strains in Taiwan [18–20], Haarlem strains (20%, 3/15), East-African Indian (EAI) strains (20%, 3/15), and Beijing strains (53.3%, 8/15) were found in this study (Fig. 2). The 32 subjects with clustered TB infection were younger (62.3, 49.1–84.3 vs. 85.8, 82.6–87.8 years, *P* < 0.01) when compared with those with individual TB infections, and other clinical variables were similar between the two groups. Interestingly, ADL-dependence and the use of nasogastric feeding, Foley, or tracheostomy, appeared to associate with TB clustered infection. Those with clustered TB infection were less likely to have nasogastric tube (25.0% vs. 90.0%, *P* < 0.01), Foley (25.0% vs. 90.0%, *P* < 0.01), and tracheostomy (28.1% vs. 60.0%, *P* = 0.07) in comparison with those with individual TB infections. In a multivariate logistic regression model adjusted for age, sex, COPD, and BMI, subjects with clustered TB infection were less likely to be in ADL-dependent status (adjusted odds ratio 0.073, 95% confidence interval 0.007–0.758) as compared with those with individual TB infections (Table 2).

**Table 1:** Characteristics of subjects at the diagnosis of pulmonary tuberculosis^a^

Characteristics	All *n* = 42	Clustered infection *n* = 32	Individual infection *n* = 10	*P* value
Demographic data				
Age (years)	**76.5 (33.8)**	**62.3 (35.2)**	**85.8 (5.1)**	*P* < 0.01
Male (%)	27 (64.3)	8 (56.2)	9 (90.0)	*P* = 0.07
Body mass index(kg/m^2^)	**20.3 (1.9)**	**20.8 (4.7)**	**19.6 (2.6)**	*P* = 0.15
Dependent activity of daily living	18 (42.9)	8 (25.0)	9 (90.0)	*P* < 0.01
Nasogastric tube feeding %	17 (40.5)	8 (25.0)	9 (90.0)	*P* < 0.01
Foley insertion %	17 (40.5)	8 (25.0)	9 (90.0)	*P* < 0.01
Tracheostomy %	15 (35.7)	9 (28.1)	6 (60.0)	*P* = 0.07
TB severity				
Cavity on chest X-ray	4 (9.5)	4 (12.5)	0(0.0)	*P* = 0.56
Positive sputum smear	6 (14.3)	5 (15.6)	1 (10.0)	*P* = 0.56
Underlying diseases				
Hypertension %	18 (42.9)	13 (40.6)	5 (50.0)	*P* = 0.72
Diabetes mellitus %	8 (19.0)	7 (21.9)	1 (10.0)	*P* = 0.66
Congestive heart failure %	7 (16.7)	5 (15.6)	2 (20.0)	*P* = 0.54
Chronic renal failure (Cr > 2) %	6 (14.3)	5 (15.6)	1 (10.0)	*P* = 0.56
Old stroke	6 (38.1)	11 (34.4)	5 (50.0)	*P* = 0.47
Chronic obstructive pulmonary disease %	8 (19.0)	4 (12.5)	4 (40.0)	*P* = 0.08
Hepatitis B virus carrier	2 (4.8)	2 (6.7)	0(0.0)	*P* = 0.56
Hepatitis C virus carrier	4 (9.5)	3 (10.0)	1 (10.0)	*P* = 0.74
Laboratory data				
White blood cell count (cells/μl)	**7140 (2680)**	**7315 (2937)**	**6930 (1178)**	*P* = 0.31
Hemoglobin (g/dL)	**11.4 (2.1)**	**11.4 (2.1)**	**11.4 (1.7)**	*P* = 0.57
Platelet (10^3^/μl)	**238 (101)**	**247 (116)**	**215 (69)**	*P* = 027
Creatinine (mg/dL)	**0.8 (0.3)**	**0.8 (0.5)**	**0.7 (0.3)**	*P* = 0.22
Albumin (mg/dL)	**3.4 (0.9)**	**3.4 (1.0)**	**3.4 (0.5)**	*P* = 0.63

^a^Data represent N(%) and **median (interquartile range).**

**Table 2:** Multivariate binary logistic regression for clustered infection

Characteristics	Univariate		Multivariate	
	*P* value	OR (95% C.I.)	*P* value	OR (95% C.I.)
Age, per 1 year increment	0.012	1.091 (1.020–1.167)	0.334	1.045 (0.956–1.141)
Sex, male	0.037	10.00 (1.151–86.876)	0.388	3.234 (0.225–46.411)
BMI, per 1 year increment(kg/m^2^)	0.194	0.857 (0.679–1.081)	0.762	0.959 (0.729–1.261)
ADL-dependence	0.002	0.030 (0.003–0.270)	0.028	0.073 (0.007–0.758)
COPD	0.038	0.176 (0.034–0.905)	0.991	1.012 (0.120–8.576)

OR Odds ratio, C.I. Confidence interval, BMI body mass index, ADL Activity of daily living, COPD Chronic obstructive pulmonary disease

### Added value of prolonged genotyping surveillance on the epidemiological investigation

**Fig. 3:**
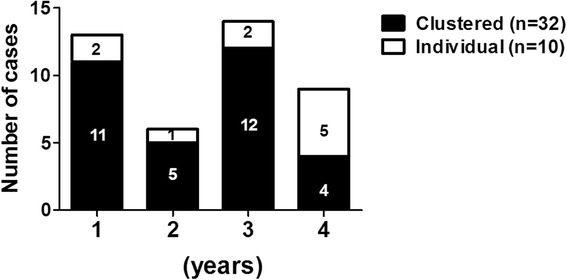
Annual data of the 42 culture-proven pulmonary tuberculosis participants classified as clustered or individual TB infection by genotyping analyses

Genotyping during the 4-year period used originally as the post-outbreak surveillance for cluster 1, not only found the other 4 clustered TB infections, but also identified 12 subjects without epidemiological linkage in cluster 1. Indeed, prolonged follow-up is essential in TB outbreak follow-up given that it may take a long time to become TB disease among patients already infected by *M. tb* [21], and genotyping is of particular use in such prolonged follow-up to differentiate those with clustered infection from coincidental TB subjects in population with high TB incidence [10]. In this study, we found the percentage of subjects with clustered infection among all TB subjects was 84.6% (11/13) in the first year, 83.3% (5/6) in the second year, 85.7% (12/14) in the third year, and 44.4% (4/9) in the fourth year (Fig. 3). Moreover, the median interval of TB diagnosis between index subject and secondary subjects was 499 (197–983) days in cluster-1, 848 days in cluster-2, 1511 (1182–1840) days in cluster-3, 217 days in cluster-4, and 107 days in cluster-5 (Table 3). Collectively, these data reflect the chronic nature of TB infection and highlights the need for prolonged genotyping surveillance to determine TB transmission.

**Table 3:** Epidemiological characteristics of the 32 subjects with clustered infection^a^

Cluster	Person	CXR	Sputum	No	Time	Place of contact	
		Cavity (+)	Smear (+)		(days)	Ward	Section
Overall	2^nd^ subjects	-	-	27	**673 (812)**	14 (43.8%)	22 (68.7%)
1	Indexsubject	Yes	Yes				
	2^nd^ subjects			22	**499 (786)**	10 (45.5%)	17 (77.3%)
2	Indexsubject	No	Yes				
	2^nd^ subject			1	848	1 (100%)	1 (100%)
3	Indexsubject	Yes	Yes				
	2^nd^ subjects			2	**1511 (329)**	2 (100%)	2 (100%)
4	Indexsubject	No	Yes				
	2^nd^ subject			1	217	0 (0%)	1 (100%)
5	Indexsubject	No	Yes				
	2^nd^ subject			1	107	1 (100%)	1 (100%)

^a^Data represent N (%) and **median (interquartile range)**

**Fig. 4:**
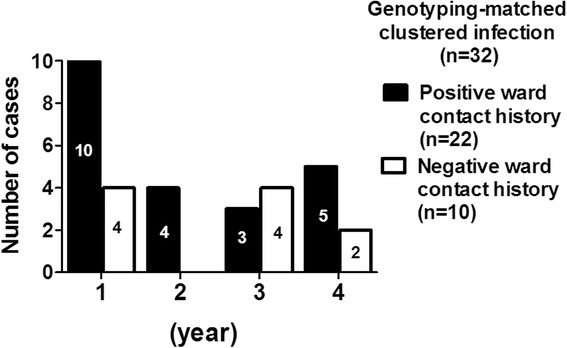
Annual data of the 32 genotyping-matched clustered infection participants by their contact history with the index subject

The epidemiological investigation alone might potentially underestimate the TB outbreak given that TB is an air-transmitted disease, and the use of genotyping has been proven to be a pivotal tool to detect epidemiologically non-linked subjects [8, 11]. In this study, we found that standard definition of TB contact, the contact in the same ward for cumulatively more than 40 h [17], only detected 68.8% (22/32) of the 32subjects with TB clustered infection. The annual data showed that the percentage of subjects with positive ward-contact history among all subjects with clustered infection was 71.4% (10/14) in the first year, 100% (4/4) in the secondary year, 42.9% (3/7) in the third year, and 71.4% (5/7) in the further year (Fig. 4). Moreover, in cluster-1 with 22 secondary TB subjects, 45.5% (10/22) of them had contact history in the same ward, 31.8% (7/22) had contact in the same facility, and 22.7% (5/22) of them did not have any contact with the index subject (seen in Table 3). These results highlight the need of regular genotyping surveillance to delineate the TB transmission in LTCFs.

## Discussion

TB outbreak in LTCFs is an important public health issue, and to delineate the route of TB transmission is particularly difficult in the population with high TB incidence. In this study, we used regular genotyping surveillance to delineate TB transmission in a complex TB outbreak consisting of 5 clusters during a 4-year follow-up. We also found that the activity of index subjects was a key factor of TB transmission, whereas epidemiological investigations had limited role given that TB is an air-transmitted disease.

Ongoing global surveillance of TB notifications has shown that high TB incidence in the elderly is an emerging worldwide problem [4, 22]. In line with Taiwan, TB notifications in Western Pacific Region and South East Asia have also found increased TB prevalence rates among people aged more than 65 years [5, 23]. LTCF scaring the elderly are generally characterized by the limited personal space and relatively enclosed environments which lead to the inevitable close and prolonged contact between residents. Residents in LTCFs are thus vulnerable to TB transmission; however, to early diagnose TB in the elderly have been found to be difficult resulting from atypical clinical manifestations of TB infection in the elderly [24, 25]. Additionally, to differentiate true outbreak from coincidental TB subjects in an aged population with high underlying TB incidence is another challenge in LTCFs. In this study, regular genotyping surveillance clearly delineated 5 clustered TB infections during the 4-year period, and our data demonstrated that clustered infection often can be identified years later after the development of TB transmission (see Table 3). Therefore, regular genotyping surveillance might be considered to be a part of post-outbreak surveillance in LTCFs.

Latent TB testing and treatment is a complex issue for the elderly in LTCFs [4]. Latent TB infection is currently majorly diagnosed by Mantoux tuberculin skin test (TST) or Interferon-gamma release assay (IGRA) [26], but age has been shown to interfere the accuracy of TST and IGRA [27, 28]. Hochberg et al. recently reported that latent TB tests in the elderly with a median age of 77 years were complicated by TST reversion and TST-IGRA discordance, possibly resulting from waning immunity in the elderly [29]. Additionally, the policy of primary Bacille Calmette − Guérin vaccination in infancy and a booster at 6–9 years of age in Taiwan further raised the concern to diagnose latent TB infection by TST alone. Moreover, 9-month Isoniazid (INH) treatment is the standard treatment for latent TB infection in Taiwan, but INH may have potential drug-interaction with medications frequently used in the elderly for medical diseases through inhibition of cytochrome P450 [30]. Therefore, latent TB diagnosis and treatment were not included in the post-outbreak management strategy in this study.

Genotyping results of this study not only to delineate clustered TB infection but also to reflect the interesting pattern of *M. tb* strains in the reported population. One recently published molecular epidemiological study found that the three major *M. tb* lineages in Taiwan including Beijing, Haarlem, and EAI strains may relate to the historical migration of different ethnic populations from China, Dutch, and Austronesian respectively [20]. The reported facility is located in southern Taiwan where Haarlem and EAI strains were predominant strains, whereas most of the subjects in Section A were veterans moved from China. Therefore, Beijing strains (53.3%, 8/15), Haarlem strains (20%, 3/15), and East-African Indian strains (20%, 3/15) were found in this study (see Fig. 2). Additionally, previous studies have reported a wide range of frequency of mixed-strain *M. tb* infection, including 11.3% in one Taiwanese study, while only one subject in cluster-5 had a mixed-strain *M.tb* infection in this study [31, 32]. Consistent with reports from Netherlands and Denmark, another unique role of genotyping in post-outbreak follow-up is to identify TB transmission happened a long time ago given that it may take a long time for those infected by *M. tb* to develop active TB disease [21, 33, 34]. As shown in this study, the median interval of diagnosis in between index subject and the 27 secondary TB subjects was 673 days (Table 3); therefore, TB transmission of a TB outbreak only can be delineated by regular genotyping surveillance.

Additionally, genotyping is increasingly used as a pivotal tool in post-outbreak surveillance to identify those without epidemiological linkages [11, 35, 36]. Epidemiological linkage, defined by contact with index subject in the same ward for cumulatively more than 40 h, represented elevated of the risk of TB infection resulting from heavy exposure in general environmental conditions [17]. In contrast to general contact conditions, LTCFs were special settings given that all of the residents were stayed in a relatively closed environment for a long time under the same central air-conditioning system. It is, therefore, unsurprising that 37.0% (10/27) of the 27 secondary TB subjects in this study had no ward-contact history with index subjects (Fig. 4). As the increased use of genotyping to identify those without epidemiological linkage with index subject, the modern contact investigation suggested incorporating non-traditional approaches including social network analysis and geographic information systems into the contact investigation of TB outbreak [37]. Such non-traditional approaches reflect the potential unrecognized TB exposure via air; however, more evidence is needed to suggest the practical approach in different settings in the future. Taken together, our findings found that the regular genotyping surveillance has a pivotal role to delineate the TB transmission in post-outbreak surveillance of the reported LTCF, and implicated the need for active case finding beyond those with ward-contact. We thought that such approach should also be applicable in similar enclosed settings including prisons or shelters.

There were limitations in this study. First, the study is a retrospective study, but the comprehensive TB outbreak management plan was established at the beginning of the outbreak management and supervised by Taiwan CDC. Second, those who died or transferred out cannot be fully surveyed; however, such potential missing reflected that the TB transmission might be more severe than we presented here with 5 clusters involving 32 patients. Third, the reported facility is a particular setting caring both the elderly and individuals with mental illness; therefore, more studies in other LTCFs are needed. Also, genotyping cannot be assessed of the six clinically diagnosed TB subjects; however, the outbreak investigation including reviewing serial images conducted by CDC showed that these six patients were less likely to be the index case.

## Conclusion

In conclusion, this study provided the evidence of added value of regular genotyping surveillance on epidemiological investigations to delineate TB transmission in the reported LTCF. We found that activity of residents is a key factor for TB transmission and demonstrated the use of regular genotyping to identify the TB-cluster without epidemiological linkages. Therefore, regular genotyping might be incorporated into the strategy to manage an outbreak of TB in LTCFs or other enclosed settings.

## Additional file

Additional file 1: Dataset of demographic and laboratory data. (XLSX 16 kb)

## Abbreviations

ADA: Adenosine aminohydrolase
ADL: Activities of daily living
BMI: Body mass index
CDC: Centers for disease control
COPD: Chronic obstructive pulmonary disease
CXR: Chest X-ray
HIV: Human immunodeficiency virus
IGRA: Interferon-gamma release assay
INH: Isoniazid
LTCFs: Long-term care facilities
*M. tb*: *Mycobacterium tuberculosis*
MIRU-VNTR: mycobacterial interspersed repetitive units-variable number tandem repeats
RFLP: Restriction fragment length polymorphism
Spoligotyping: Spacer oligonucleotide typing
TB: Tuberculosis
TST: Tuberculin skin test
UPGMA: Unweighted pair group method with arithmetic averages
